# Supplementary figures and images for: Modernising tactile acuity assessment; clinimetrics of semi-automated tests and effects of age, sex and anthropometry on performance
Source: PeerJ. 2021 Oct 25;9:e12192. doi: 10.7717/peerj.12192 (PMC8759376; doi:10.7717/peerj.12192)

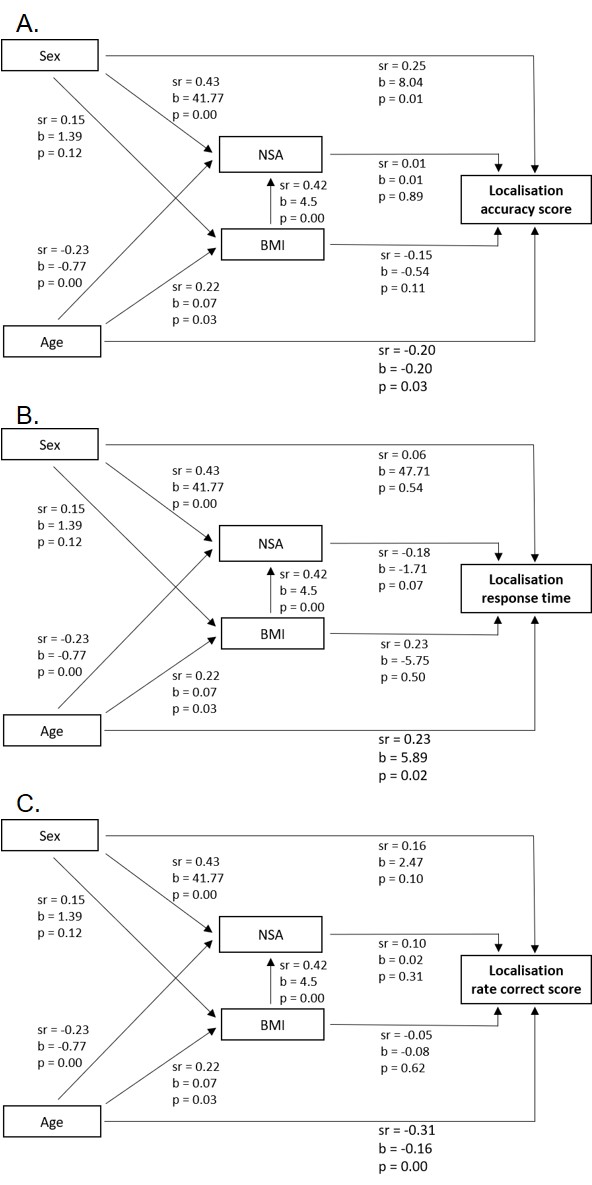

Supplement: Supplemental Information 1 — Relationships between age, sex (female =0; male =1), body mass index (BMI), neck surface area (NSA) and iTAD localisation scores from the sequential mediation analyses. Relationships are expressed in semi-partial correlations (sr) and unstandardized regression coefficients (b), including their level of significance (p). In all models, BMI and NSA did not significantly (p > 0.05) mediate the relationship between either age or sex with iTAD scores. Models provided for: localisation accuracy score (A), response time (B) and rate correct score (C). [file peerj-09-12192-s001.jpg]

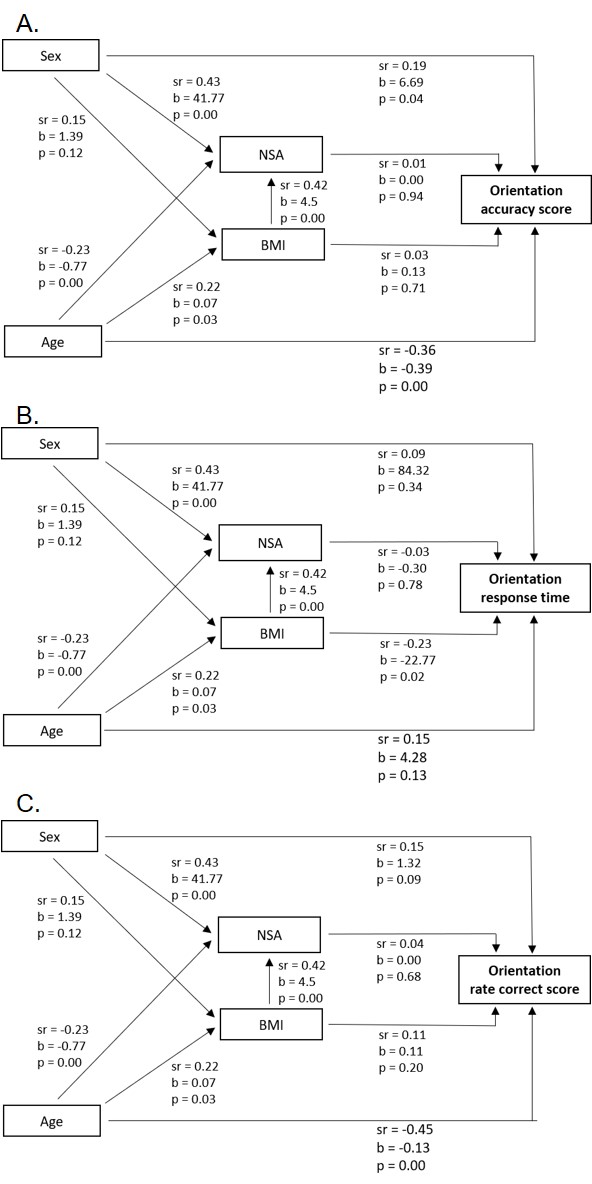

Supplement: Supplemental Information 2 — Relationships between age, sex (female =0; male =1), body mass index (BMI), neck surface area (NSA) and iTAD orientation scores from the sequential mediation analyses. Relationships are expressed in semi-partial correlations (sr) and unstandardized regression coefficients (b), including their level of significance (p). In all models, BMI and NSA did not significantly (p > 0.05) mediate the relationship between either age or sex with iTAD scores. Models provided for: orientation accuracy score (A), response time (B) and rate correct score (C). [file peerj-09-12192-s002.jpg]

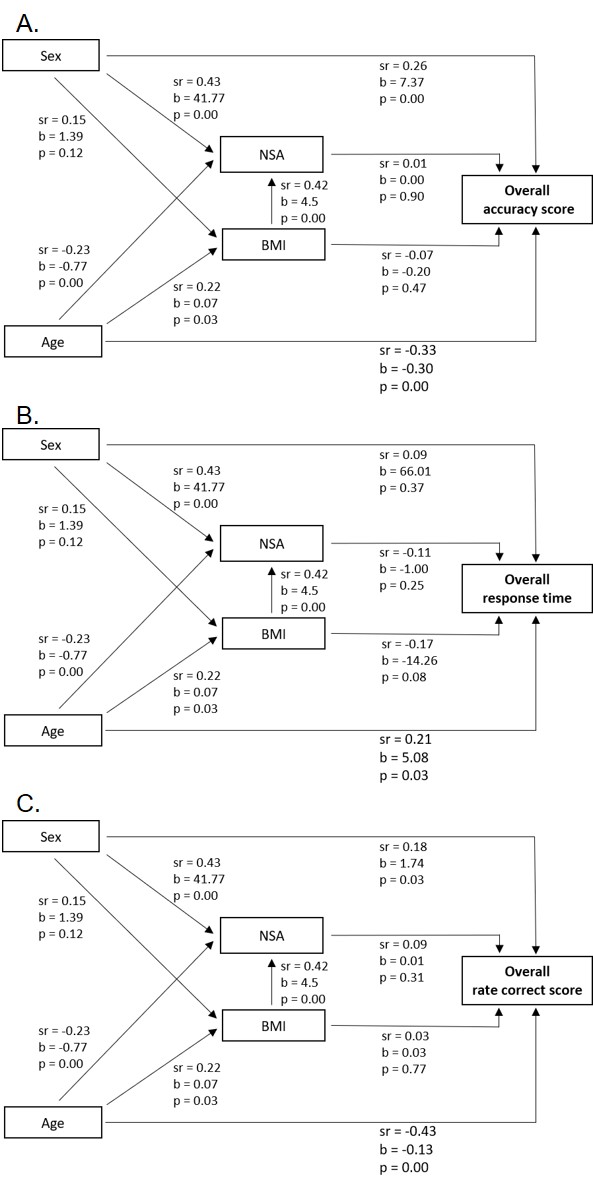

Supplement: Supplemental Information 3 — Relationships between age, sex (female =0; male =1), body mass index (BMI), neck surface area (NSA) and iTAD overall scores from the sequential mediation analyses. Relationships are expressed in semi-partial correlations (sr) and unstandardized regression coefficients (b), including their level of significance (p). In all models, BMI and NSA did not significantly (p > 0.05) mediate the relationship between either age or sex with iTAD scores. Models provided for: overall accuracy score (A), response time (B) and rate correct score (C). [file peerj-09-12192-s003.jpg]

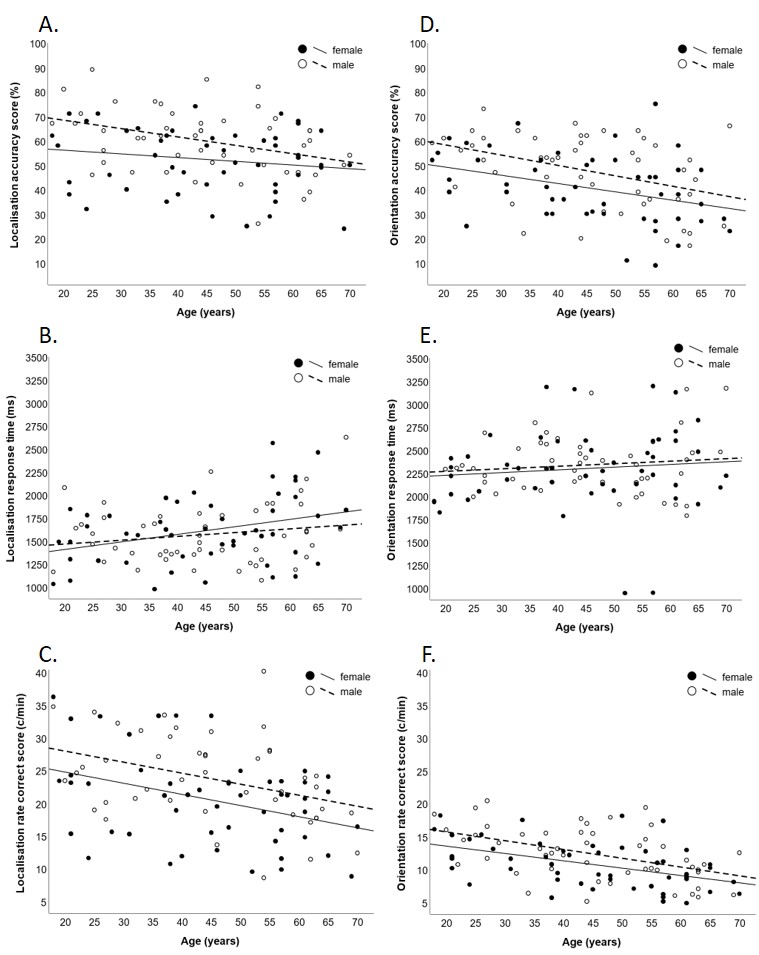

Supplement: Supplemental Information 4 — Scatter plots of iTAD scores as a function of age and sex. Scores are displayed for localisation accuracy (A), response time (B), and rate correct score (C), as well as orientation accuracy (D), response time (E), and rate correct score (F). Lines represent the least squares regressions. ms, milliseconds; c/min, correct responses per minute. [file peerj-09-12192-s004.jpg]

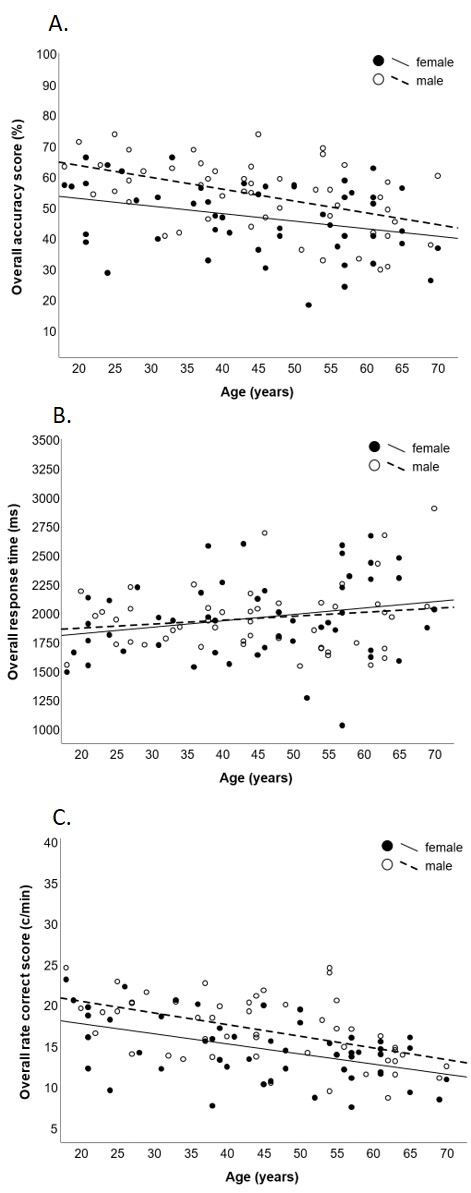

Supplement: Supplemental Information 5 — Scatter plots of iTAD overall scores (mean of localisation and orientation scores) as a function of age and sex. Scores are displayed for overall accuracy (A), response time (B), and rate correct score (C). Lines represent the least squares regressions. ms, milliseconds; c/min, correct responses per minute. [file peerj-09-12192-s005.jpg]
